# Drivers of Macrofungi Community Structure Differ between Soil and Rotten-Wood Substrates in a Temperate Mountain Forest in China

**DOI:** 10.3389/fmicb.2018.00037

**Published:** 2018-01-23

**Authors:** Yun Chen, Jens-Christian Svenning, Xueying Wang, Ruofan Cao, Zhiliang Yuan, Yongzhong Ye

**Affiliations:** ^1^College of Forestry, Henan Agricultural University, Zhengzhou, China; ^2^Center for Biodiversity Dynamics in a Changing World (BIOCHANGE), Aarhus University, Aarhus, Denmark; ^3^Section for Ecoinformatics and Biodiversity, Department of Bioscience, Aarhus University, Aarhus, Denmark; ^4^College of Life Sciences, Henan Agricultural University, Zhengzhou, China

**Keywords:** forest dynamics plot, forest canopy, plant–macrofungi relationship, dispersal limitation, species protection, stand structure, temperate forest

## Abstract

The effects of environmental and dispersal processes on macrofungi community assembly remain unclear. Further, it is not well understood if community assembly differs for different functional guilds of macrofungi, e.g., soil and rotten-wood macrofungi. In this study, using 2433 macrofungi sporocarps belonging to 217 species located within a forest dynamics plot in temperate mountain forest (China), we examined the explanatory power of topography, spatial eigenvectors (representing unknown spatial processes, e.g., dispersal), plant community, and light availability for local spatial variation in the macrofungi community through variance partitioning and partial least squares path modeling. We found spatial eigenvectors and light as the most important factors for explaining species richness and composition of macrofungi. Light was negatively correlated with species richness of macrofungi. Furthermore, species richness and composition of soil macrofungi were best explained by light, and species richness and composition of rotten-wood macrofungi were best explained by spatial eigenvectors. Woody plant community structure was not an important factor for species richness and composition of macrofungi. Our findings suggest that spatial processes, perhaps dispersal limitation, and light availability were the most important factors affecting macrofungi community in temperate deciduous broad-leaved forest. Major differences in influencing factors between soil and rotten-wood macrofungi were observed, with light as the major driver for soil macrofungi and unknown spatial processes as the major driver for rotten-wood macrofungi. These findings shed new light to the processes shaping community assembly in macrofungi in temperate deciduous broad-leaved forest and point to the potential importance of both intrinsic dynamics, such as dispersal, and external forcing, such as forest dynamics, via its effect on light availability.

## Introduction

Macrofungi exert profound biological and economic impacts (Miles and Chang, 2004). The main macrofungi includes ascomycetes and basidiomycetes with large, easily observed spore-bearing structures. They constitute an important part of terrestrial ecosystems, forming a large share of their species diversity, and are key players in ecosystem processes (Senn-Irlet et al., 2007). For example, macrofungi are involved in biomass decomposition (Miles and Chang, 2004) and can rapidly migrate and colonize new areas faster than some vascular plants (Alday et al., 2017). Despite the importance of macrofungi in nutrient cycling and succession in forest ecosystems, our current understanding of species diversity, community structure, and dynamics in macrofungi remains limited (Ferrer and Gilbert, 2003). The evaluation of the structure and dynamics of a macrofungi community and factors driving its variability provides information for future sustainable management of the diversity of macrofungi (Senn-Irlet et al., 2007).

Conservation awareness and measures of macrofungi resources are largely lacking (Senn-Irlet et al., 2007). Some macrofungi are at risk of extinction because of human interference and environmental damage (Senn-Irlet et al., 2007). Identification of major drivers of macrofungi community assembly is thus a crucial step to conserve their diversity (Chen et al., 2017). Ecologists conducted numerous studies on major factors affecting plant and animal community. However, community assembly in macrofungi is poorly studied and understood due to their largely hidden nature and often short-lived sporocarps (Senn-Irlet et al., 2007). Rainfall and temperature are important factors for interannual variation in macrofungi community (Ágreda et al., 2016). Spatial studies on macrofungi have mainly focused on describing species diversity in different types of forests (Nordén and Paltto, 2001) and determining their relationship to plant community (Ferrer and Gilbert, 2003) and topography (Alday et al., 2017). Furthermore, the relationships between wood-decay and rotten-wood fungi have been widely investigated (Nordén and Paltto, 2001; Blaser et al., 2013; Kwaśna et al., 2017). Although these studies may contribute to our understanding of macrofungi community assembly, the major drivers of macrofungi community remain unclear.

Ecological drift and dispersal probably are important drivers for community dynamics of woody plants, in supplement to environmental filtering (Legendre et al., 2009). Dispersal limitation has been shown to shape microorganism communities, such as arbuscular mycorrhizal fungi, soil ascomycetous fungi, bacteria, and ectomycorrizas (Gao et al., 2015; Stegen et al., 2015). Macrofungi that are reproduced by spore dispersal may be limited by suitability of habitats (Rolstad et al., 2004). In recent years, neutral theory has been increasingly used to explain community assembly mechanisms (Hubbell, 2006). Neutral theory states that within guilds, plant or microorganism species have equal competitiveness, and species distributions are only affected by dispersal limitation and demographic stochasticity (Hubbell, 2006; Jia et al., 2015). The role of dispersal in macrofungi community assembly should be investigated by excluding the influences of environmental factors (Gao et al., 2015; Chen et al., 2016). However, the relative importance of environmental processes and dispersal processes in macrofungi community assembly has been rarely investigated.

The growth of most fungi is related to light, and strong light may inhibit or even kill mycelia (Miles and Chang, 2004). Forest canopy creates vertical gradients of light, temperature, and vapor pressure deficit (Nakamura et al., 2017). Forest canopy is an important factor affecting the dynamics of forest ecosystems and habitat formation (Nakamura et al., 2017). Thomaes et al. (2013) showed that trees promote or hinder the growth and distribution of understory species. Barbier et al. (2008) reported that trees affect understory species under changing light conditions. The structural characteristics of forest canopy remarkably vary monthly, particularly in temperate deciduous broad-leaved forests (Miles and Chang, 2004). The change of light condition has important influence on atmospheric temperature and humidity (Svrcek and Coxon, 1975). Moreover, light may be related to the growth of macrofungi. Thus, light may have direct or indirect effects on the assembly of macrofungi communities. However, the relationship between light and macrofungi remains unclear. To our knowledge, few scholars have reported the effect of light on macrofungi.

Rotten wood is an important habitat that can sustain the growth of macrofungi (Blaser et al., 2013). Therefore, many more studies on rotten-wood macrofungi are available compared to those of macrofungi that grow on other substrates (Nordén and Paltto, 2001; Blaser et al., 2013; Kwaśna et al., 2017). However, the characteristics of growth and distribution may differ among fungi growing on different substrates (Senn-Irlet et al., 2007), and the drivers of community structure in rotten wood relative to soil macrofungi have not been well elucidated yet.

Temperate mountain forests, widely distributed in northern and eastern China, are dominated by deciduous trees and show wide variation in community structure in time and space scales. Macrofungi are species rich in these ecosystems (Senn-Irlet et al., 2007). In this study, we conducted a monthly collection of all macrofungi in a 5 ha forest dynamics plot from the beginning of May to the end of October 2016. This study aimed to do the following: (1) identify the effects of environmental and dispersal processes on macrofungi community assembly in temperate deciduous broad-leaved forest; and (2) assess if differences in the drivers between soil and rotten-wood macrofungi in the examined temperate mountain forest exist.

## Materials and Methods

### Study Area

The Baiyunshan National Nature Reserve (BNNR), with an area of approximately 168 km^2^, is located in Songxian County in the extreme north of Henan Province, East China (111°48′–112°16′E, 33°33′–33°56′N). The reserve was set up in the temperate zone in 1982 to preserve a portion of the deciduous broad-leaved forest in the region. The reserve includes 2404 known species of vascular plants, belonging to 815 genera and 170 families. A total of 17 species are included in the Chinese list of rare and endangered species. The annual mean temperature in the region is 13.5°C, the annual mean precipitation is 1200 mm, and forest cover is 81.2%. The dominant tree species in the deciduous broad-leaved forest and in the plot are *Quercus aliena* var. *acuteserrata, Toxicodendron vernicifluum*, and *Sorbus alnifolia*.

### Sampling Design

In 2015, a permanent plot covering 5 ha (250 m × 200 m, horizontal distance) was established within the deciduous broad-leaved forest in the BNNR. Data were collected following the plot standards of the Center for Tropical Forest Science network (Condit, 1995). All trees with a diameter at breast height ≥ 1 cm were tagged, identified, and measured during the summer of 2015. The plot was rugged, that is, the elevation varied from 1538 to 1600 m above sea level, and the 20-m cell slopes varied from 4.3 to 55.5°. The plot contains 17,963 individual trees belonging to 55 families and 93 species.

All macrofungi sporocarps in the 5 ha plot were collected based on the 20 m × 20 m subplots monthly from the beginning of May to the end of October 2016. Macrofungi began to grow when the weather became warm in early summer (May) and disappeared when the weather became cold in late autumn (October) in this temperate area. Macrofungi were investigated six times at the end of each month, and substrate types, including rotten wood, living tree, soil, and litterfall, were recorded. Each month, all macrofungi were counted and harvested from each of the 20 m × 20 m subplots. Before specimen collection, the collectors underwent professional training with regard to the basic characteristics of macrofungi and different methods of collection in the field.

In the laboratory, the macrofungi sporocarps collected were identified mainly based on macroscopic morphology (including the external shape, color, velum, prosthecae, and mediotrastum) and microscopic characteristics (including the basidiospore, basidium, cystidium, and trama). Initial identification was applied to genus or subgenus level based on macroscopic morphology. The microscopic characteristics were observed under the microscope by using freehand slicing. Then, identification was applied to species level through consultation with appropriate literature (Mao, 2000). A few sporocarps could not be identified to the species level. Hence, we consulted experts on species classification and referred to the reaction characteristics of some chemical agents (Mao, 2000). A list of macrofungi species names are shown in Supplementary Table S1.

### Topography, Light, Plant Community, and Spatial Data

Four topographic attributes were measured for each 20 m × 20 m subplot in the field, namely, elevation (with values from 1538 to 1600 m), slope (with values from 4.31 to 55.50°), aspect (with values from 2.17 to 3.07), and convex and concave (with values from -45.72 to 59.26°) (**Table 1**). These parameters were measured following the methods described by Harms et al. (2001) and Valencia et al. (2004). An aspect is a circular variable; sin (aspect) and cos (aspect) were computed to use aspect in models.

**Table 1:** Overviews of macrofungi, topographical factors, stand structure, and light in the Baiyunshan forest plot.

Variables	Minimum	Average value (±*SD*)	Maximum
Fungi species richness	0.000	9.269 ± 5.263	24.000
Slope (°)	4.311	25.450 ± 8.820	55.503
Aspect	2.173	2.697 ± 0.1539	3.066
Elevation (m)	1538.000	1570.000 ± 15.282	1600.000
Convex concave (°)	-45.726	1.172 ± 14.118	59.260
Stand density	29.000	138.200 ± 69.372	512.000
Basal area (cm^2^)	21.530	302.440 ± 177.644	931.030
Woody plant richness	7.000	16.140 ± 5.245	40.000
Woody plant diversity	0.581	0.818 ± 0.068	0.948
Leaf area index	1.433	1.873 ± 0.181	2.358
Average leaf angle (°)	20.680	42.550 ± 0.181	65.620
Canopy cover	0.484	0.661 ± 0.055	0.763
Total radiation [mol/(m^2^-d)]	2894	3613 ± 505.897	5256
Scattered radiation [mol/(m^2^-d)]	424.500	513.000 ± 43.663	643.700
Direct radiation [mol/(m^2^-d)]	2465.000	3613.000 ± 479.332	5256.000
Light transmittance	0.124	0.186 ± 0.025	0.259

Light availability of the forest canopy at the study site was evaluated. Each 20 m × 20 m subplot in the plot was divided into four 10 m × 10 m subplots. Hemispherical photographs were taken at the center of each 10 m × 10 m subplot at 1.3 m aboveground by using a Canon 7D camera (Kyushu, Japan) monthly from May to October 2016. Three replicate photos were taken, and photos showing the highest contrast between sky and foliage for each 10 m × 10 m subplot were selected. The camera was equipped with an ultra-wide-angle fisheye lens (Fukushima-ken, Japan) arranged horizontally. Data were collected early in the morning, during late dusk, or on overcast days whenever possible to avoid inaccurate readings caused by direct sunlight (Han et al., 2017). Finally, the Gap Light Analyzer software (version 2.0) was used to measure single leaf area index, canopy cover, average leaf angle, scattered radiation, direct radiation, and transmittance of light (Han et al., 2017). Light and macrofungi were simultaneously investigated. The mean value derived from six surveys of the forest canopy was used for analysis. Principal component (PC) analysis was applied using the RDA command in the VEGAN package to reduce the seven light variables to a convenient number of predictors (Oksanen et al., 2007). The first four light PCs (largely reflecting variables in parentheses) explained 99.0% of the total variation of light variables: PC1 (canopy gap and radiation), 44.7%; PC2 (canopy cover), 34.3%; PC3 (light transmittance), 11.3%; and PC4 (scattered radiation), 8.6% (Supplementary Table S2).

A total of 93 woody plant species were previously identified. Of the 93 woody plant species, *Q. aliena* var. *acuteserrata* is the dominant species (accounting for 27.87% of total plant basal area). PC analysis was applied to reduce the basal areas of the 93 plant species into a convenient number of predictors, which was transformed by “Hellinger.” The first six PCs (largely reflecting variables in parentheses) explained 80.14% of the total variation of plant basal area: PC1 (basal area of *Q. aliena* var. *acuteserrata*), 42.1%; PC2 (basal area of *Pinus armandii*), 13.6%; PC3 (basal area of *P. tabuliformis*), 9.6%; PC4 (basal area of *T. vernicifluum* and *Euptelea pleiosperma*), 6.3%; PC5 (basal area of *Lindera obtusiloba* and *S. alnifolia*), 5.0%; and PC6 (basal area of *Corylus heterophylla*), 3.3% (Supplementary Table S3).

Principal coordinates of neighbor matrices (PCNM) were used to determine spatial eigenvectors by using the method reported by Borcard and Legendre (2002) and Legendre et al. (2009). PCNM eigenvectors can reflect the importance of spatial processes. A total of 91 PCNM eigenvectors were generated and subjected to forward selection (with permutation tests, at the 0.05 significance level). Significant PCNM eigenvectors were analyzed, and the model was simplified (Supplementary Figure S1). PCNM were performed using the PCNM package.

### Statistical Procedures

Variance partitioning (Legendre et al., 2009) can be used to evaluate the effects of environmental and spatial processes on community ecology. Variation of richness and species compositions in the overall soil and rotten-wood macrofungi were partitioned among the topographical factors (elevation, slop, aspect, and convex and concave), plant community (plants PC1–PC6), light (lights PC1–PC4), and spatial (PCNM vectors) eigenvectors by using the VARPART function in the VEGAN package (Oksanen et al., 2007).

Forest ecological systems are complex and are characterized by multiple interacting processes. Target variables, such as species richness, may be influenced directly and indirectly by a set of variables. In contrast to ordination analysis and variance partitioning, partial least squares path modeling (PLS-PM) allows the user to determine the indirect effects of two explanatory variables (Sanchez, 2013; Hodapp et al., 2015; Musseau et al., 2015). PLS-PM is a robust form of structural equation modeling that is more efficient than covariance method (Hodapp et al., 2015; Musseau et al., 2015). The first step of the PLS-PM analysis consists of pooling correlated observed variables in blocks. Each block is a latent variable. The latent variables are connected to each other by links called “paths.” A path coefficient represents a direct effect of a latent variable on another one. Total effects are the sum of the direct and indirect effects. The full path model includes an inner model (the relationship between latent variables) and an outer model (the relationship among indicators) (Hodapp et al., 2015; Musseau et al., 2015). In the present study, PLS-PM was used to explore the causal relationships among macrofungi species richness or species compositions, topographical factors (elevation, slop, aspect, and convex and concave), plant community (plants PC1–PC6), light (lights PC1–PC4), and spatial eigenvectors (PCNM vectors). On the basis of the theoretical knowledge and results of the variance partitioning, a conceptual model was established in which topographical factors, plant community, light, and spatial eigenvectors affect the macrofungi species diversity or richness. PLS-PM is a complex method, and additional details on the methodology and application of this method are described in the extensive work of Hodapp et al. (2015). PLS-PM was performed using the PLSPM package (Sanchez, 2013). All analyses were conducted in R 3.4.0 (R Development Core Team^1^).

## Results

### Species Composition and Community Dynamics

**FIGURE 1:**
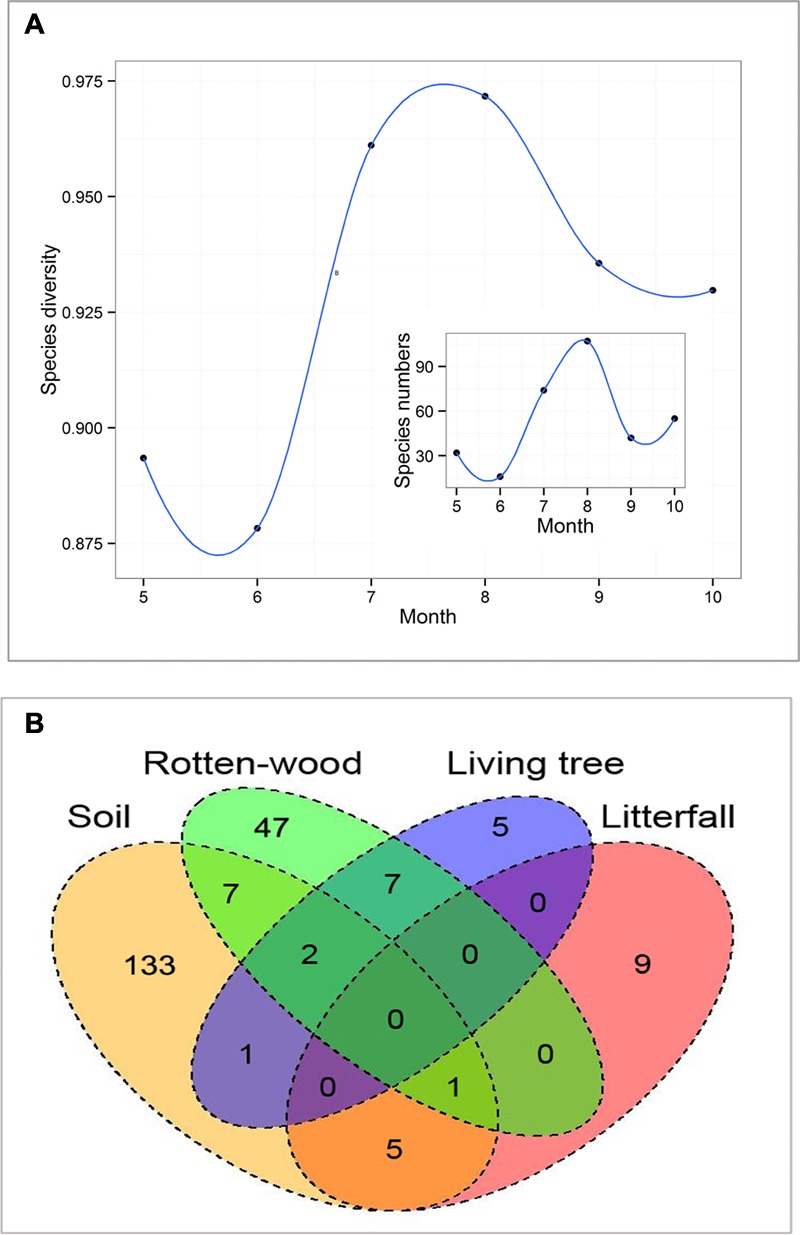
Species diversity patterns of macrofungi under different months **(A)**. The subset shows the species richness patterns of macrofungi along the months. Species diversity was measured by Simpson’s Diversity Index. Species richness is the number of species. **(B)** It shows the number of macrofungi species in different substrates.

We recorded 2433 macrofungi sporocarps from 217 species in 33 families between May and October 2016. The species richness of macrofungi hump-shaped distributed from May to October. The species richness and composition of macrofungi remarkably differed in the different months (**Figure 1A**). Moreover, the species diversity of macrofungi differs among different substrates, and 89.4% species tended to be distributed in one kind of substrate (**Figure 1B**). A total of 149, 64, 15, and 15 macrofungi species were recorded in the soil, rotten wood, living tree, and litterfall, respectively. The most abundant macrofungi species were soil macrofungi, followed by rotten-wood macrofungi. The spatial distribution characteristics of the overall soil and rotten-wood macrofungi are shown in Supplementary Figure S2.

### Effect of Environmental Factors on Macrofungi Community

**FIGURE 2:**
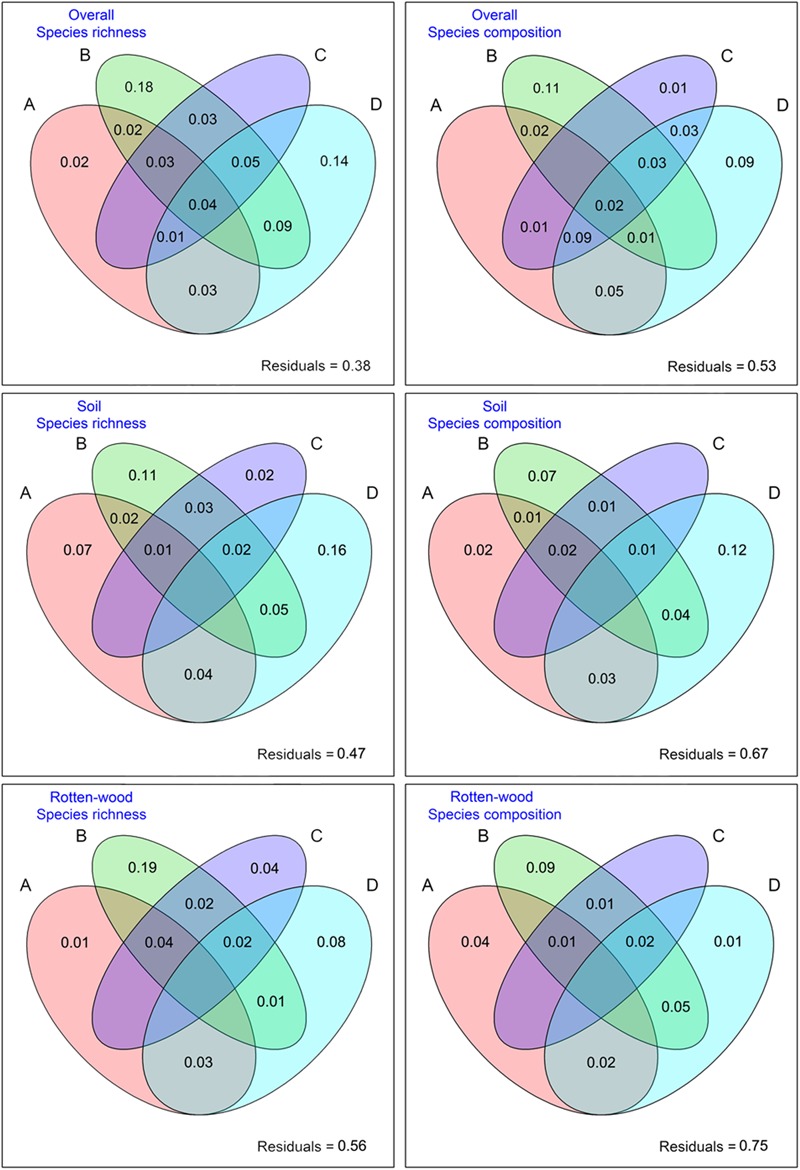
Variance partitioning of the effects of topography **(A)**, spatial eigenvectors **(B)**, plant community **(C)**, and light **(D)** on the species richness and composition of all macrofungi (first row) and of rotten wood (second row) and soil fungi (third row). Values less than zero are not shown. Topographical factors **(A)**: elevation, slop, aspect, and convex and concave. Spatial eigenvectors **(B)**: PCNM vectors. Plant community **(C)**: plants PC1–PC6. Light **(D)**: light PC1–PC4. Spatial eigenvectors were obtained by PCNM.

Variation partitioning showed that topography, spatial eigenvectors, plant community, and light explained 62.0 and 47.0% of the variation in species richness and composition for the overall macrofungi, respectively (**Figure 2**). For the macrofungi species richness, 18.0% of the variation was attributed to pure spatial eigenvectors, and 14.0% was attributed to pure light. For macrofungi species composition, 11.0% of the variation could be attributed to pure spatial eigenvectors, and 9.0% was attributed to pure light.

**FIGURE 3:**
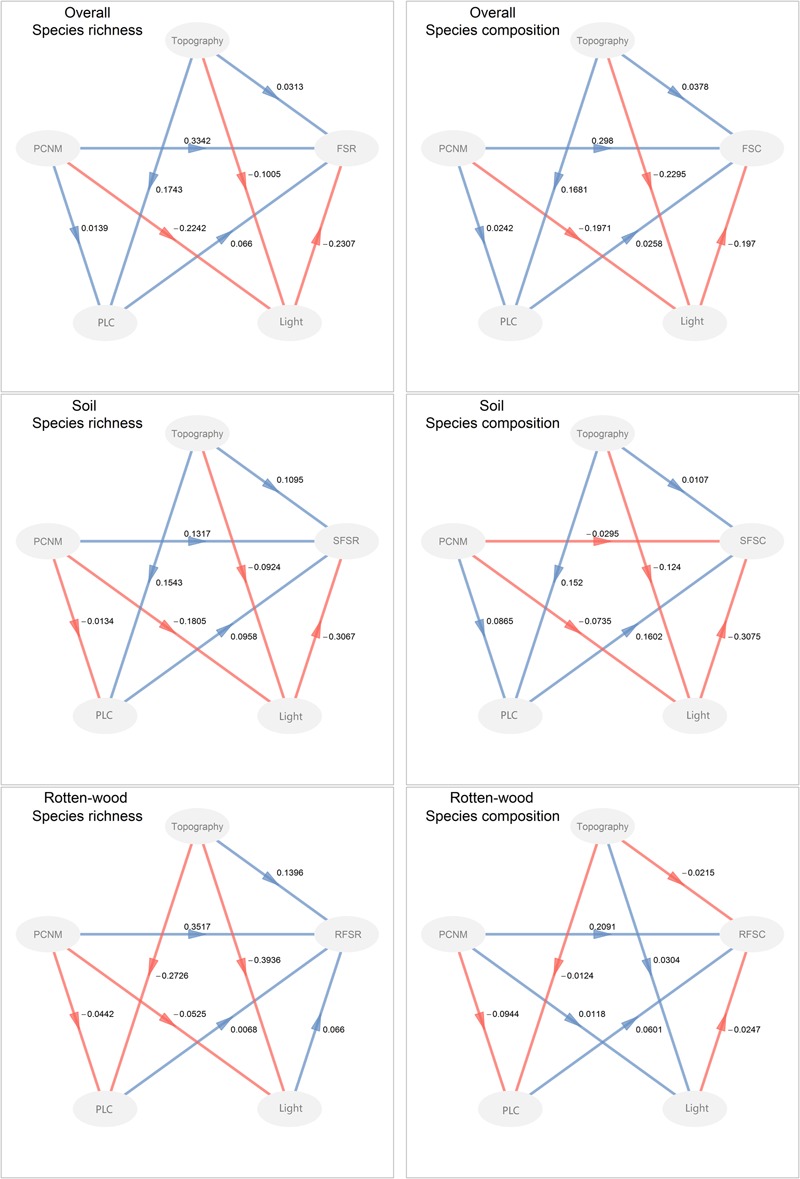
Partial least squares path modeling (PLS-PM) of the effects of topography, spatial eigenvectors (PCNM), plant community (PLC), and light on the overall fungi species richness (FSR), overall fungi species composition (FSC), soil fungi species richness (SFSR), soil fungi species composition (SFSC), rotten-wood fungi species richness (RFSR), and rotten-wood fungi species composition (RFSC). The numbers above the arrows indicate path coefficients. The path coefficients above the arrows indicate the direct effect of a latent variable on another one. Blue and red lines indicate positive and negative pathways, respectively. The outer models (relationships among indicators) of PLS-PM are illustrated in Supplementary Figure S3. The standardized direct, indirect, and total effects of topography, PCNM eigenvectors, plant community, and light on the species richness and composition of overall soil and rotten-wood macrofungi are illustrated in Supplementary Table S3.

The PLS-PM results indicated that the two highest path coefficients were for PCNM eigenvectors and the light was for macrofungi species richness and composition (**Figure 3**). For macrofungi species richness, the path coefficients of PCNM eigenvectors and light were 33.42 and -23.07%, respectively. For macrofungi species composition, the path coefficients of PCNM eigenvectors and light were 29.80 and -19.70%, respectively.

### Difference between Soil and Rotten-Wood Macrofungi

For species richness, the analysis of variation partitioning showed that all environmental factors explained 53.0 and 44.0% of the variations in the soil and rotten-wood macrofungi, respectively (**Figure 2**). For species richness of soil macrofungi, the effects of pure light (16.0%) were higher than those of pure spatial eigenvectors (11.0%). For species richness of rotten-wood macrofungi, the effects of pure spatial eigenvectors (19.0%) were higher than those of pure light (8.0%). For species composition, the analysis of variation partitioning showed that all environmental factors explained 33.0 and 25.0% of the variations in the soil and rotten-wood macrofungi, respectively (**Figure 2**). For the species composition of soil macrofungi, the effects of pure light (12.0%) are higher than those of pure spatial eigenvectors (7.0%). For the species composition of rotten-wood macrofungi, the effects of pure spatial eigenvectors are the highest among the factors (9.0%).

The PLS-PM results indicated that the path coefficients of light (30.67%) are higher than those of PCNM eigenvectors (13.17%) for species richness of soil macrofungi (**Figure 3**). For species richness of rotten-wood macrofungi, the path coefficients of PCNM variables (35.17%) are higher than those of light (6.60%). For species composition of soil macrofungi, the path coefficients of light are the highest (30.75%). For species composition of rotten-wood macrofungi, the path coefficients of PCNM eigenvectors are the highest (20.91%). The outer models (relationships among indicators) of PLS-PM are illustrated in Supplementary Figure S3. The standardized direct, indirect, and total effects of topography, PCNM eigenvectors, plant community, and light on the species richness and composition of overall soil and rotten-wood macrofungi are illustrated in Supplementary Table S4.

## Discussion

The present study took an important step forward toward the understanding of the assembly mechanisms of macrofungi community, with respect to species richness and composition of macrofungi in temperate deciduous broad-leaved forest. Furthermore, dispersal limitation and light availability could be the major drivers of the species richness and composition of macrofungi in temperate deciduous broad-leaved forest. The major influencing factors differ between soil and rotten-wood macrofungi community.

### Major Drivers of Macrofungi Community Structure

Variation in the species richness and compositions of macrofungi is mainly explained by light and spatial eigenvectors. In particular, light availability was negatively correlated with the species richness and composition of macrofungi. This conclusion is supported by Sysouphanthong et al. (2010). Strong light facilitates water evaporation, and the humidity level is high under dense canopy cover (Svrcek and Coxon, 1975). Moreover, humus is prevalent under low-light conditions (Miles and Chang, 2004). Therefore, macrofungi species may not prefer high-light habitats.

In this study, spatial eigenvectors were considered an important factor affecting the species richness and species composition of the overall macrofungi. Spatial eigenvectors exhibited high explanatory power after controlling for the effects of topography, plant community, and light by variance partitioning. The PLS-PM also indicated the significant effect of spatial eigenvectors on the species richness and composition of overall macrofungi. The dispersability of macrofungi and the suitability of habitat are two important conditions for the successful colonization of macrofungi (Rolstad et al., 2004; Jönsson et al., 2008). Studies on spore dispersal indicated that some fungi can disperse far from the fruiting body, although the vast majority of spore falls within a few meters (Jönsson et al., 2008). Edman et al. (2004) reported that local dispersal sources strongly affect the colonization patterns of rotten-wood fungi, and the mass deposition of spores may be necessary for successful colonization. Abrego et al. (2015) indicated that macrofungi are sensitive to habitat loss and fragmentation. Therefore, dispersal limitation may be an important factor affecting the species richness and composition of the overall macrofungi. However, spatial eigenvectors measured are not exactly equal to the dispersal capabilities of fungal species. Many scholars have conducted active exploration in the dispersal capabilities of fungi (Jönsson et al., 2008). This study will help us further understand the effects of the spatial process on fungi community assembly. However, spatial eigenvectors might also at least partially represent unmeasured environmental or biotic factors. Temperature and precipitation are the two major determinants of macrofungi (Zhai and Pan, 2003; Piao et al., 2006). Macrofungi are also affected by the chemical properties of soil (Rühling and Tyler, 1990; O’Hanlon and Harrington, 2012), the chemical properties and structural characters of rotten wood (Blaser et al., 2013; Kwaśna et al., 2017), and the age of forest (Stokland et al., 1997; Nordén and Paltto, 2001). The consideration of more environmental factors will be more meaningful, because some factors may together significantly contribute to macrofungi community variance.

In forest ecosystems, topography is the most common factor that determines distinct species communities (Shetie et al., 2017; Wright et al., 2017). However, our results showed that the explanatory power of topographic factors is weak, like reflecting relatively limited topographic variability within the study site.

Previous studies found that woody plants are an important factor for the species composition of macrofungi (Ferrer and Gilbert, 2003). With increasing woody plant diversity, the rate of successful colonization may increase because plants provide various types of habitats for macrofungi (Ferrer and Gilbert, 2003). Unexpectedly, the explanatory power of woody plant community is weak in our study site. These findings are consistent with the report by Vacher et al. (2008), who showed that plant species diversity is not a major determinant of fungi richness. This condition may be because few macrofungi grow on woody plants within the study site. Soil and rotten-wood macrofungi are the major species comprising macrofungi. Thus, the woody plant community is not a factor that determines species richness and composition of macrofungi in the examined temperate mountain forest.

### Difference in the Major Drivers between Soil and Rotten-Wood Macrofungi

Macrofungi species in the plot were categorized into two main groups, namely, soil and rotten-wood fungi, to determine their major influencing factors. Light is the major driver for soil macrofungi, and spatial eigenvectors are the major driver for rotten-wood macrofungi. This finding could be caused by a random and fragment distribution of rotten wood (Fridman and Walheim, 2000; Harmon, 2001). Rotten wood is an important structural and functional component of forest ecosystems (Rolstad et al., 2004), and dead wood sustains the growth of wood-living organisms (Martikainen et al., 2000). Dispersal can be a limiting factor for the occurrence of wood-decaying fungi, perhaps reflecting the fragmented occurrence of this substrate type (Rolstad et al., 2004; Jönsson et al., 2008). Substrate of soil macrofungi is continuous in space and time, allowing easy spread. Thus, the effect of dispersal limitation on soil fungus should be smaller than on rotten-wood macrofungi, which is in agreement with our results. However, macrofungi need a suitable habitat for colonization on the soil, and environmental spatial heterogeneity becomes a strongly limiting factor for macrofungi distribution on the ground. A number of fungi require light for the normal development of the fruiting body, and ultraviolet light in the region of 200 to 300 nm affects the vegetative growth of fungi (Miles and Chang, 2004). Therefore, light is one of the limiting factors for soil macrofungi species.

## Conclusion and Implications

Spatial processes, perhaps dispersal limitation, and light availability were the most important factors affecting macrofungi community in temperate deciduous broad-leaved forest. Woody plant community structure was not an important factor for species richness and composition of macrofungi in this study. Major differences in influencing factors between soil and rotten-wood macrofungi were observed, with light as the major driver for soil macrofungi and unknown spatial processes as the major driver for rotten-wood macrofungi. These findings shed new light to the processes shaping community assembly in macrofungi in temperate deciduous broad-leaved forest and point to the potential importance of both intrinsic dynamics, such as dispersal, and external forcing, such as forest dynamics, via its effect on light availability.

This study elucidated the factors influencing macrofungi community structure and provided an opportunity to improve biodiversity conservation in temperate deciduous broad-leaved forest for sustainable forest management (Chen et al., 2017). Our findings suggest that macrofungi should be treated with different protection strategies because their major drivers differ between soil and rotten-wood macrofungi community in temperate deciduous broad-leaved forest. Macrofungi do not prefer high-light habitats, and dead wood in the forest must be retained. Dead wood can provide continuous and diversified spaces to sustain the growth of macrofungi and reduce the dispersal limitation. However, macrofungi slightly changes from year to year even in the same forest, although an outline of macrofungi succession and richness seems to be recognizable from this observation in a year. Many new species may be expected in additional years. Therefore, long-term monitoring of the macrofungi would be necessary in future studies. In addition, different macrofungi species may have different growth habits, and their correlations with environmental factors also differ. If some macrofungi species are more prone to disturbances, then they could act as reporter species for forest fitness. Therefore, individual fungal species also are the object of our attention in future studies.

## Author Contributions

YY conceived the ideas. YC developed the methodology. YC and J-CS led the writing of the manuscript. YC, XW, RC, and ZY conducted the fieldwork.

## Conflict of Interest Statement

The authors declare that the research was conducted in the absence of any commercial or financial relationships that could be construed as a potential conflict of interest.

## Footnotes

1 http://www.Rproject.org
