# Supplementary material for: Drivers of Macrofungi Community Structure Differ between Soil and Rotten-Wood Substrates in a Temperate Mountain Forest in China
Source: Front Microbiol. 2018 Jan 23;9:37. doi: 10.3389/fmicb.2018.00037 (PMC5787090; doi:10.3389/fmicb.2018.00037)
Supplement: Supplementary file 1 [file Data_Sheet_1.pdf]

Table S1 A list of all identified macrofungi species in Baiyunshan plot

Species	Family	Species	Family
Daedalea biennis	*Polyporaceae*	*Lactarius deliciosus*	*Russulaceae*
Agaricus abruptibulbus	*Tricholomataceae*	*Lactarius fuliginosus*	*Russulaceae*
Agaricus comtulus	*Tricholomataceae*	*Lactarius gerardii*	*Russulaceae*
Agaricus placomyces	*Tricholomataceae*	*Lactarius glyciosmus*	*Russulaceae*
Agaricus silvaticus	*Tricholomataceae*	*Lactarius hatsudake*	*Russulaceae*
Agaricus silvicola	*Tricholomataceae*	*Lactarius pallidus*	*Russulaceae*
Albatrellus dispansus	*Polyporaceae*	*Lactarius piperatus*	*Russulaceae*
Aleurodiscus amorphus	*Amanitaceae*	*Lactarius sanguifluus*	*Russulaceae*
Amanita caesarea	*Amanitaceae*	*Laetiporus sulphureus*	*Polyporaceae*
Amanita ceciliae	*Amanitaceae*	*Leccinum griseum*	*Boletaceae*
Amanita excelsa	*Amanitaceae*	*Leccinum scabrum*	*Boletaceae*
Amanita fulva	*Amanitaceae*	*Lentinu edodes*	*Pleurotaceae*
Amanita citrina	*Amanitaceae*	*Lenzites betulina*	*Polyporaceae*
Amanita subjunquillea	*Amanitaceae*	*Lepista nuda*	*Tricholomataceae*
Amanita pantherina	*Amanitaceae*	*Lepista personata*	*Tricholomataceae*
Amanita sculpta	*Amanitaceae*	*Leucocoprinus birnbaumii*	*Psathyrellaceae*
Amanita spissa	*Amanitaceae*	*Limacella glioderma*	*Amanitaceae*
Amanita spissacea	*Amanitaceae*	*Lycoperdon perlatum*	*Lycoperdaceae*
Amanita spreta	*Amanitaceae*	*Marasmius cohaerens*	*Tricholomataceae*
Amanita parvipantherina	*Amanitaceae*	*Marasmius epiphyllus*	*Tricholomataceae*
Amanita verna	*Amanitaceae*	*Marasmius maximus*	*Tricholomataceae*
Amanita virosa	*Amanitaceae*	*Marasmius neosessilis*	*Tricholomataceae*
Armillaria luteo-virens	*Tricholomataceae*	*Marasmius oreades*	*Tricholomataceae*
Armillariella mellea	*Tricholomataceae*	*Marasmius siccus*	*Tricholomataceae*
Asterophora lycoperdoides	*Tricholomataceae*	*Melanoleuca strictipes*	*Tricholomataceae*
Auriscalpium vulgare	*Auriscalpiaceae*	*Melanoleuca cognata*	*Tricholomataceae*
Bjerkandera fumosa	*Polyporaceae*	*Melanoleuca exscissa*	*Tricholomataceae*
Bolbitius vitellinus	*Bolbitiaceae*	*Polyporus alveolaris*	*Polyporaceae*
Boletellus longicollis	*Strobilomycetaceae*	*Merulius tremellosus*	*Polyporaceae*
Boletus albus	*Boletaceae*	*Mycena citrinella*	*Tricholomataceae*
Boletus edulis	*Boletaceae*	*Mycena galericulata*	*Tricholomataceae*
Boletus flammans	*Boletaceae*	*Mycena lactea*	*Tricholomataceae*
Boletus impolitus	*Boletaceae*	*Mycoleptodonoi des aitchisonii*	*Clinacodontaceae*
Boletus luridus	*Boletaceae*	*Naematoloma fasciculare*	*Strophariaceae*
Boletus magnificus	*Boletaceae*	*Naematoloma sublateritium*	*Strophariaceae*
Boletus ornatipes	*Boletaceae*	*Odontia queletii*	*Hydnaceae*
Boletus pseudocalopus	*Boletaceae*	*Oligoporus caesius*	*Polyporaceae*
Boletus pulverulentus	*Boletaceae*	*Oligoporus tephroleucus*	*Polyporaceae*
Boletus retipes	*Boletaceae*	*Oudemansiella mucida*	*Tricholomataceae*
Boletus speciosus	*Boletaceae*	*Oudemansiella platyphylla*	*Tricholomataceae*
Boletus zelleri	*Boletaceae*	*Phellinus pini*	*Polyporaceae*
Dacrymyces palmatus	*Dacrymycetaceae*	*Phellinus gilvus*	*Polyporaceae*

Cantharellus cibarius	*Cantharellaceae*	*Pholiota nameko*	*Strophariaceae*
Cantharellus infundibuliformis	*Cantharellaceae*	*Pleurocybella porrigens*	*Pleurotaceae*
Cantharellus lateritius	*Cantharellaceae*	*Pleurotus cystidiosus*	*Pleurotaceae*
Geastrum saccatum	*Geastraceae*	*Pluteus cervinus*	*Pluteaceae*
Ramaria fumigata	*Ramariaceae*	*Pluteus pantherinus*	*Pluteaceae*
Clitocybe odora	*Tricholomataceae*	*Pluteus longistriatus*	*Pluteaceae*
Clitopilus prunulus	*Tricholomataceae*	*Polyporus elegans*	*Polyporaceae*
Collybia acervata	*Tricholomataceae*	*Porodisculus pendulus*	*Polyporaceae*
Tricholomopsis platyphylla	*Tricholomataceae*	*Psathyrella campestris*	*Psathyrellaceae*
Collybia dryophlia	*Tricholomataceae*	*Psathyrella candolleana*	*Psathyrellaceae*
Conocybe lactea	*Bolbitiaceae*	*Psathyrella subinceta*	*Psathyrellaceae*
Coprinus plicatilis	*Psathyrellaceae*	*Psathyrella velutina*	*Psathyrellaceae*
Polystictus membranaceus	*Polyporaceae*	*Psathvrella spadiceogrisea*	*Psathyrellaceae*
Coriolus consors	*Polyporaceae*	*Pycnoporus cinnabarinus*	*Polyporaceae*
Coriolus unicolor	*Polyporaceae*	*Pycnoporus coccineus*	*Polyporaceae*
Coriolus versicolor	*Polyporaceae*	*Ramaria botrytoides*	*Ramariaceae*
Cortinarius crocolitus	*Cortinariaceae*	*Ramaria obtusissima*	*Ramariaceae*
Cortinarius largus	*Cortinariaceae*	*Rhodophyllus clypeatus*	*Entolomataceae*
Crepidotus mollis	*Basidiomycetes*	*Rhodophyllus lazulinus*	*Entolomataceae*
Cryptoporus volvatus	*Polyporaceae*	*Rhodophyllus prunuloides*	*Entolomataceae*
Cyclomyces fuscus	*Polyporaceae*	*Rhodophyllus rhodopolius*	*Entolomataceae*
Cystoderma granulosum	*Agaricaceae*	*Russula albida*	*Russulaceae*
Daedalea dickinsii	*Polyporaceae*	*Russula aurata*	*Russulaceae*
Dictyopanus pusillus	*Pleurotaceae*	*Russula aquosa*	*Russulaceae*
Panus rudis	*Pleurotaceae*	*Russula cyanoxantha*	*Russulaceae*
Favolus alveolaris	*Polyporaceae*	*Russula decolorans*	*Russulaceae*
Favolus arcularius	*Polyporaceae*	*Russula delica*	*Russulaceae*
Favolus mollis	*Polyporaceae*	*Russula flavida*	*Russulaceae*
Flammulina velutiper	*Tricholomataceae*	*Russula foetens*	*Russulaceae*
Fomitopsis pinicola	*Polyporaceae*	*Russula heterophylla*	*Russulaceae*
Fomitopsis vinosa	*Polyporaceae*	*Russula luteolacta*	*Russulaceae*
Geastrum velutinum	*Geastraceae*	*Russula nigricans*	*Russulaceae*
Gerronema fibula	*Tricholomataceae*	*Russula olivacea*	*Russulaceae*
Gloeophyllum sepiarium	*Polyporaceae*	*Russula pectinaoides*	*Russulaceae*
Gomphidius roseus	*Gomphidiaceae*	*Russula rosea*	*Russulaceae*
Grifola fronaosa	*Polyporaceae*	*Russula rubra*	*Russulaceae*
Gyroporus purpurinus	*Boletaceae*	*Russula sororia*	*Russulaceae*
Hirschioporus lacteus	*Polyporaceae*	*Russula vesca*	*Russulaceae*
Hydnum repandum	*Hydnaceae*	*Russula vinosa*	*Russulaceae*
Hygrocybe cantharellus	*Hygrophoraceae*	*Russula violeipes*	*Russulaceae*
Hygrophorus miniatus	*Hygrophoraceae*	*Russula virescens*	*Russulaceae*
Hygrocybe punicea	*Hygrophoraceae*	*Scleroderma areolatum*	*Lycoperdaceae*
Hygrophorus ceraceus	*Hygrophoraceae*	*Scutellinia scutellata*	*Pezizaceae*
Hygrophorus lucorum	*Hygrophoraceae*	*Gyrophana lacrymans*	*Polyporaceae*

Hypholoma cinnabarinum	*Psathyrellaceae*	*Sirobasidium magnum*	*Sirobasidiaceae*
Hypocrea argillacea	*Hypocreaceae*	*Steccherinum ochraceum*	*Steccheriaceae*
Inocybe asterospora	*Cortinariaceae*	*Stereopsis burtianum*	*Stereaceae*
Inocybe umbrinella	*Cortinariaceae*	*Stereum gausapatum*	*Stereaceae*
Inonotus sinensis	*Polyporaceae*	*Stereum ostrea*	*Stereaceae*
Ischnoderma resinosum	*Polyporaceae*	*Stereum rugosum*	*Stereaceae*
Laccaria amethystea	*Tricholomataceae*	*Strobilomyces confusus*	*Strobilomycetaceae*
Laccaria laccata	*Tricholomataceae*	*Strobilomyces seminudus*	*Strobilomycetaceae*
Laccaria proxima	*Tricholomataceae*	*Strobilurus strobilaceus*	*Strobilomycetaceae*
Laccaria vinaceoavellanea	*Tricholomataceae*	*Suillus luteus*	*Boletaceae*
Lactarius akahatus	*Russulaceae*	*Cyptotrama chrysopeplum*	*Tricholomataceae*
Lactarius camphoratus	*Russulaceae*	*Termitomyces albuminosus*	*Tricholomataceae*
Lactarius controversus	*Russulaceae*	*Thelephora aurantiotincta*	*Thelephoraceae*
Trametes gibbosa	*Polyporaceae*	*Thelephora caryophellea*	*Thelephoraceae*
Trametes griseo-dura	*Polyporaceae*	*Tylopilus virens*	*Boletaceae*
Trametes orientalis	*Polyporaceae*	*Tyromyces chioneus*	*Boletaceae*
Tricholoma flavovirens	*Tricholomataceae*	*Volvariella bombycina*	*Pluteaceae*
Tricholoma matsutake	*Tricholomataceae*	*Volvariella pusilla*	*Pluteaceae*
Tricholoma terreum	*Tricholomataceae*	*Volvariella speciosa*	*Pluteaceae*
Tylopilus felleus	*Boletaceae*	*Volvariella volvacea*	*Pluteaceae*
Tylopilus plumbeoviolaceus	*Boletaceae*	*Xerocomus badius*	*Boletaceae*
Russula decipiens	*Russulaceae*	*Xeromphalina tenuipes*	*Tricholomataceae*
Russula compacta	*Russulaceae*		

Table S2 Principal component analysis (PCA) of light factors

Light factors	PC1	PC2	PC3	PC4
Leaf area index	1.792	0.684	-0.305	-0.686
Average leaf angle (°)	1.232	1.589	0.293	-0.185
Canopy cover	-0.543	-1.883	-0.604	-0.049
Total radiation (mol/(m^2-d))	-1.627	1.053	-0.627	-0.379
Scattered radiation (mol/(m^2-d))	-1.372	1.143	0.634	0.823
Direct radiation (mol/(m^2-d))	-1.592	1.007	-0.719	-0.475
Light transmittance	1.152	0.590	-1.248	1.030
Eigenvalue	3.125	2.403	0.794	0.608
Proportion explained	0.447	0.343	0.113	0.086
Accumulative proportion	0.447	0.789	0.903	0.990

Table S3 Principal component analysis (PCA) of basal area of plant species

Plant species	PC1	PC2	PC3	PC4	PC5	PC6
Euonymus maackii	0.009	0.000	0.003	0.003	-0.006	0.007
Betula platyphylla	0.941	-0.537	0.790	-1.928	0.203	-0.151
Fraxinus chinensis	0.012	-0.008	0.016	-0.005	-0.023	-0.006
Symplocos paniculata	-0.395	0.266	0.121	0.111	0.272	0.383
Ribes pachysandroides	-0.003	-0.003	0.001	0.001	0.000	-0.003
Ailanthus altissima	-0.004	-0.002	0.001	0.000	-0.005	0.000
Evodia fargesii	0.011	0.000	0.004	0.003	-0.007	0.009
Evodia daniellii	0.102	-0.059	0.060	-0.064	0.024	-0.031
Maddenia hypoleuca	0.003	-0.006	-0.017	0.005	-0.004	-0.008
Meliosma flexuosa	0.060	-0.002	0.027	0.014	-0.011	0.005
Aralia elata	0.088	-0.048	0.042	-0.074	0.033	0.066
Cornus controversa	0.006	-0.012	-0.022	0.012	0.010	0.024
Quercus serrata var. *brevipetiolata*	0.186	-0.113	0.168	0.165	-0.034	-0.150
Cerasus polytricha	0.082	-0.070	-0.124	0.010	-0.014	0.032
Carpinus turczaninowii	-0.001	-0.060	0.167	-0.007	0.077	-0.251
Abelia uniflora	-0.002	-0.003	0.000	-0.001	0.005	0.009
Lonicera hispida	0.000	-0.008	-0.017	-0.001	-0.003	-0.010
Acer davidii subsp. *grosseri*	0.079	-0.048	0.053	0.077	0.032	-0.029
Malus honanensis	-0.198	-0.125	0.164	-0.026	-0.246	0.151
Ulmus davidiana	0.002	-0.001	0.001	0.003	-0.001	0.000
Lespedeza bicolor	0.005	-0.002	0.004	-0.006	-0.002	-0.003
Malus hupehensis	0.021	-0.004	0.000	-0.008	-0.003	0.003
Sorbus hupehensis	0.065	0.036	0.063	0.067	-0.194	-0.067
Crataegus hupehensis	0.068	0.004	0.033	0.073	-0.057	-0.006
Lonicera tatarinowii	-0.002	-0.001	0.001	0.002	-0.002	0.000
Tilia japonica	0.021	0.106	0.010	-0.053	-0.079	-0.020
Pinus armandii	0.978	6.082	-0.774	-0.515	0.230	0.107
Corylus chinensis	0.448	-0.026	0.301	0.085	0.058	-0.383
Viburnum betulifolium	0.036	-0.002	-0.212	-0.053	0.001	0.005
Cotoneaster acutifolius	0.033	-0.001	0.018	-0.016	-0.009	0.017
Cornus schindleri subsp. *poliophylla*	-0.004	-0.005	0.002	0.002	-0.002	0.006
Viburnum opulus var. *sargentii*	0.041	-0.023	0.003	-0.011	0.006	-0.025
Betula chinensis	-0.003	0.067	-0.008	-0.021	0.004	0.009
Sambucus williamsii	0.009	0.001	0.005	0.006	-0.009	0.001
Cornus walteri	-0.003	0.013	-0.001	-0.007	0.003	0.003
Diospyros lotus	0.003	-0.002	0.001	0.003	0.003	-0.002
Cornus macrophylla	-0.004	-0.017	0.044	0.059	-0.055	-0.015
Styrax hemsleyanus	0.017	-0.010	0.025	-0.023	-0.001	0.054
Forsythia suspensa	-0.206	-0.074	0.135	-0.146	0.162	0.373
Cercidiphyllum japonicum	0.009	-0.003	0.007	-0.011	-0.003	-0.006
Betula luminifera	0.506	-0.296	0.202	-0.571	0.149	-0.096
Padus buergeriana	0.003	0.000	0.001	0.001	-0.002	0.002

Euptelea pleiosperma	1.980	-0.928	1.152	-2.188	0.539	-1.124
Abelia biflora	-0.008	-0.023	0.029	0.027	-0.046	-0.018
Malus kansuensis	0.003	0.000	0.001	0.001	-0.002	0.002
Koelreuteria paniculata	0.000	0.000	0.000	0.000	-0.001	0.000
Lespedeza buergeri	0.002	-0.010	-0.021	-0.008	-0.004	0.001
Cornus walteri	-0.005	-0.006	0.003	0.004	-0.005	-0.003
Berberis mitifolia	0.004	0.000	0.002	0.002	-0.003	0.003
Celastrus orbiculatus	0.042	-0.054	-0.046	0.051	0.039	0.013
Elaeagnus umbellata	0.008	0.004	0.005	0.010	0.011	-0.008
Meliosma veitchiorum	0.238	-0.099	0.138	0.274	-0.100	0.133
Toxicodendron vernicifluum	1.561	-0.084	-0.315	2.252	2.318	-1.015
Carpinus cordata	1.026	-0.513	0.696	0.768	0.228	-0.592
Fraxinus paxiana	-0.002	0.002	0.000	-0.001	0.001	-0.001
Buckleya graebneriana	-0.001	-0.002	0.002	-0.001	-0.002	-0.002
Litsea tsinlingensis	0.137	0.018	0.184	0.445	-0.092	-0.034
Berberis circumserrata	0.010	0.055	-0.014	0.003	-0.005	-0.011
Acer davidii	0.039	-0.012	0.091	0.066	-0.133	0.097
Quercus aliena var. *acutiserrata*	-10.770	0.044	-0.308	-0.167	0.399	-0.491
Lindera obtusiloba	0.583	-0.130	0.924	1.924	-1.780	0.136
Elaeagnus angustifolia	0.005	-0.005	0.006	0.007	-0.006	0.005
Sinowilsonia henryi	0.034	-0.001	0.011	0.011	-0.021	0.027
Malus baccata	0.000	-0.002	0.001	0.003	-0.002	0.001
Philadelphus incanus	-0.028	-0.052	0.026	0.062	-0.022	0.101
Populus davidiana	0.007	0.028	0.004	0.016	0.004	0.011
Cerasus serrulata	0.002	0.004	0.002	0.014	0.016	-0.024
Crataegus pinnatifida	0.020	0.013	0.007	0.013	0.010	0.010
Euonymus schensianus	-0.002	0.041	0.013	-0.004	-0.048	-0.007
Tilia paucicostata	0.059	0.021	0.071	0.021	-0.161	0.038
Spiraea hirsuta	0.002	-0.002	-0.004	0.001	0.002	0.002
Euonymus phellomanus	0.000	-0.002	0.002	0.001	-0.001	-0.001
Quercus variabilis	0.068	0.028	0.022	0.086	0.010	0.083
Fraxinus mandshurica	0.014	-0.005	0.008	-0.009	0.000	-0.006
Cotoneaster multiflorus	0.000	0.000	0.001	-0.001	-0.001	-0.002
Sorbus alnifolia	-0.312	-0.053	0.387	-0.036	-1.705	0.244
Cornus kousa subsp. *chinensis*	-0.342	-0.017	0.250	0.213	-0.347	0.465
Ostrya japonica	0.035	-0.016	0.020	-0.020	0.007	-0.018
Cerasus clarofolia	0.165	-0.121	0.090	0.107	-0.210	0.137
Acer pictum subsp. mono	0.048	-0.073	0.078	0.075	-0.197	-0.019
Deutzia parviflora	0.001	0.001	0.001	0.000	-0.004	0.001
Lonicera microphylla	0.002	0.000	0.001	0.001	-0.001	0.002
Acer griseum	0.013	0.000	0.010	0.033	0.015	-0.039
Salix floderusii	0.074	-0.037	0.057	-0.145	0.010	-0.066
Juglans cathayensis	0.616	-0.355	0.401	-0.025	0.062	0.200
Pinus tabuliformis	1.337	-1.332	-4.966	-0.350	-0.591	-0.373

Yulania denudata	0.016	0.046	0.002	-0.005	-0.014	0.003
Styrax obassis	0.012	0.022	0.019	0.024	-0.036	-0.012
Acer truncatum	0.203	-0.119	0.088	0.176	0.133	-0.149
Rhododendron micranthum	-0.136	-0.073	-0.107	-0.458	-0.321	-0.088
Sorbaria sorbifolia	0.155	-0.087	-0.039	-0.164	0.021	-0.125
Corylus heterophylla	0.080	-1.072	-0.314	-0.236	1.551	2.475
Stachyurus chinensis	0.018	-0.006	0.014	-0.023	-0.007	-0.011
Eigenvalue	306.147	99.216	70.407	46.100	36.665	24.235
Proportion explained	0.421	0.136	0.096	0.063	0.050	0.033
Accumulative proportion	0.421	0.557	0.654	0.717	0.768	0.801

Table S4 Standardized direct, indirect and total effects of topography, spatial eigenvectors (PCNM), plant community, and light on overall fungi species richness, overall fungi species composition, soil fungi species richness, soil fungi species composition, rotten-wood fungi species richness, and rotten-wood fungi species composition, as determined by partial least squares path modeling (PLS-PM). Spatial eigenvectors are represented by PCNM eigenvectors

Variables	Species richness			Species composition		
	Direct path	Indirect path	Total effect	Direct path	Indirect path	Total effect
Overall						
Topography	0.0313	0.0347	0.0660	0.0378	0.0495	0.0873
PCNM	0.3342	0.0527	0.3869	0.2980	0.0395	0.3374
Plant	0.0660	0.0000	0.0660	0.0258	0.0000	0.0258
Light	-0.2307	0.0000	-0.2307	-0.1970	0.0000	-0.1970
Soil						
Topography	0.1095	0.0431	0.1526	0.0107	0.0625	0.0732
PCNM	0.1317	0.0541	0.1857	-0.0295	0.0365	0.0069
Plant	0.0958	0.0000	0.0958	0.1602	0.0000	0.1602
Light	-0.3067	0.0000	-0.3067	-0.3075	0.0000	-0.3075
Rotten-wood						
Topography	0.1396	-0.0278	0.1118	-0.0215	-0.0015	-0.0230
PCNM	0.3517	-0.0038	0.3479	0.2091	-0.0060	0.2031
Plant	0.0068	0.0000	0.0068	0.0601	0.0000	0.0601
Light	0.0660	0.0000	0.0660	-0.0247	0.0000	-0.0247

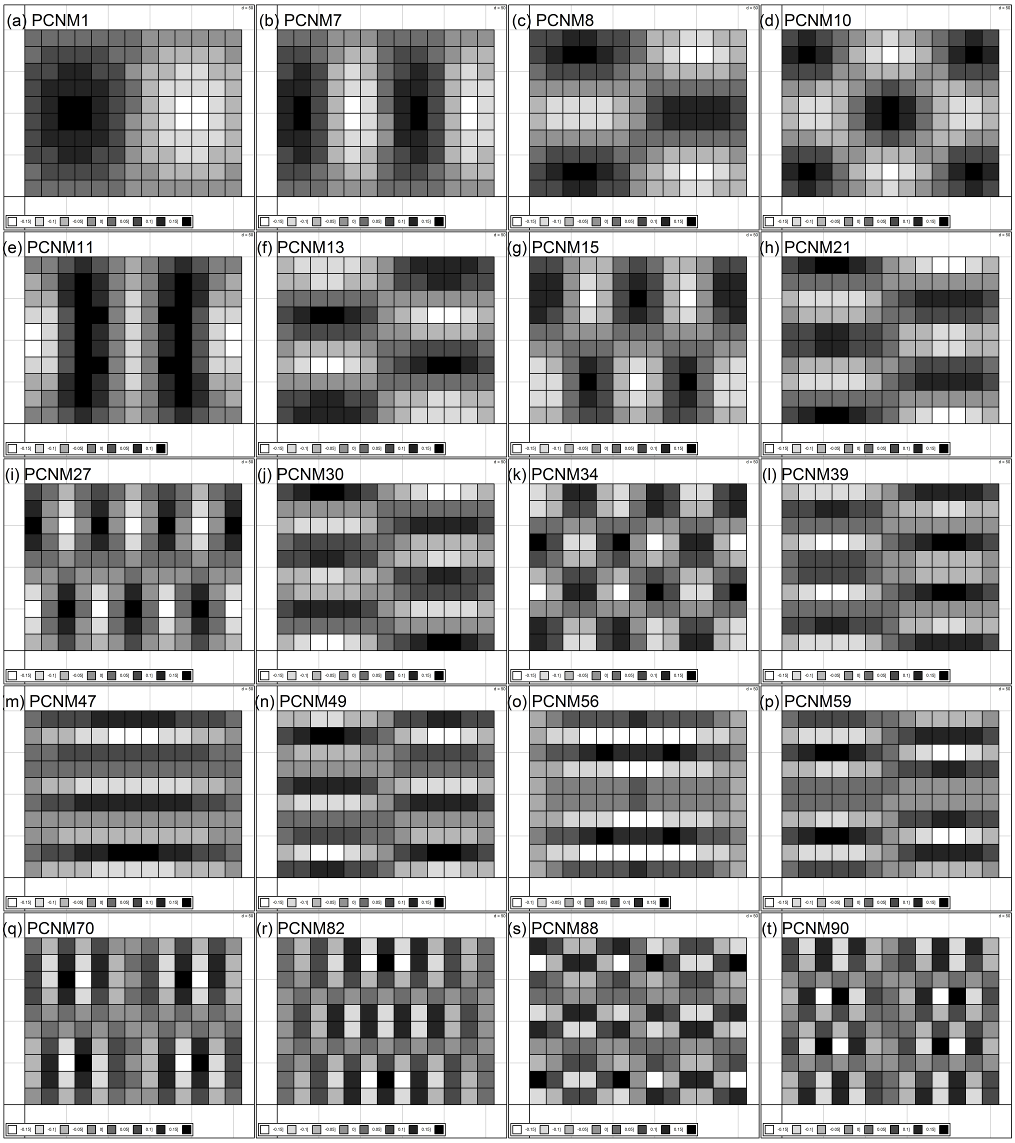

Fig. S1 Spatial distribution of spatial eigenvectors. Spatial eigenvectors were obtained by principal coordinates of neighbor matrices (PCNM). Significant spatial eigenvectors were forward selected before the analysis (*P*<0.05). Ninety-one spatial PCNM eigenvectors were created based on the 5 ha forest plot. PCNM1 (a), PCNM7 (b), PCNM8 (c), PCNM11 (e), PCNM15 (g), and PCNM21 (h) were significantly correlated with overall macrofungi species richness. PCNM1 (a), PCNM8 (c), PCNM21 (h), PCNM27 (i), PCNM30 (j), and PCNM59 (p) were significantly correlated with overall macrofungi species composition. PCNM1 (a), PCNM7 (b), PCNM8 (c), PCNM10 (d), PCNM15 (g), and PCNM21 (h) were significantly correlated with soil macrofungi species richness. PCNM13 (f), PCNM21 (h), PCNM30 (j), PCNM39 (l), and PCNM70 (q) were significantly correlated with soil macrofungi species composition. PCNM21 (h), PCNM47 (m), PCNM49 (n), PCNM82 (r), PCNM88 (s), and PCNM90 (t) were significantly correlated with rotten-wood macrofungi species richness. PCNM34 (k) and PCNM56 (o) were significantly correlated with rotten-wood macrofungi species composition.

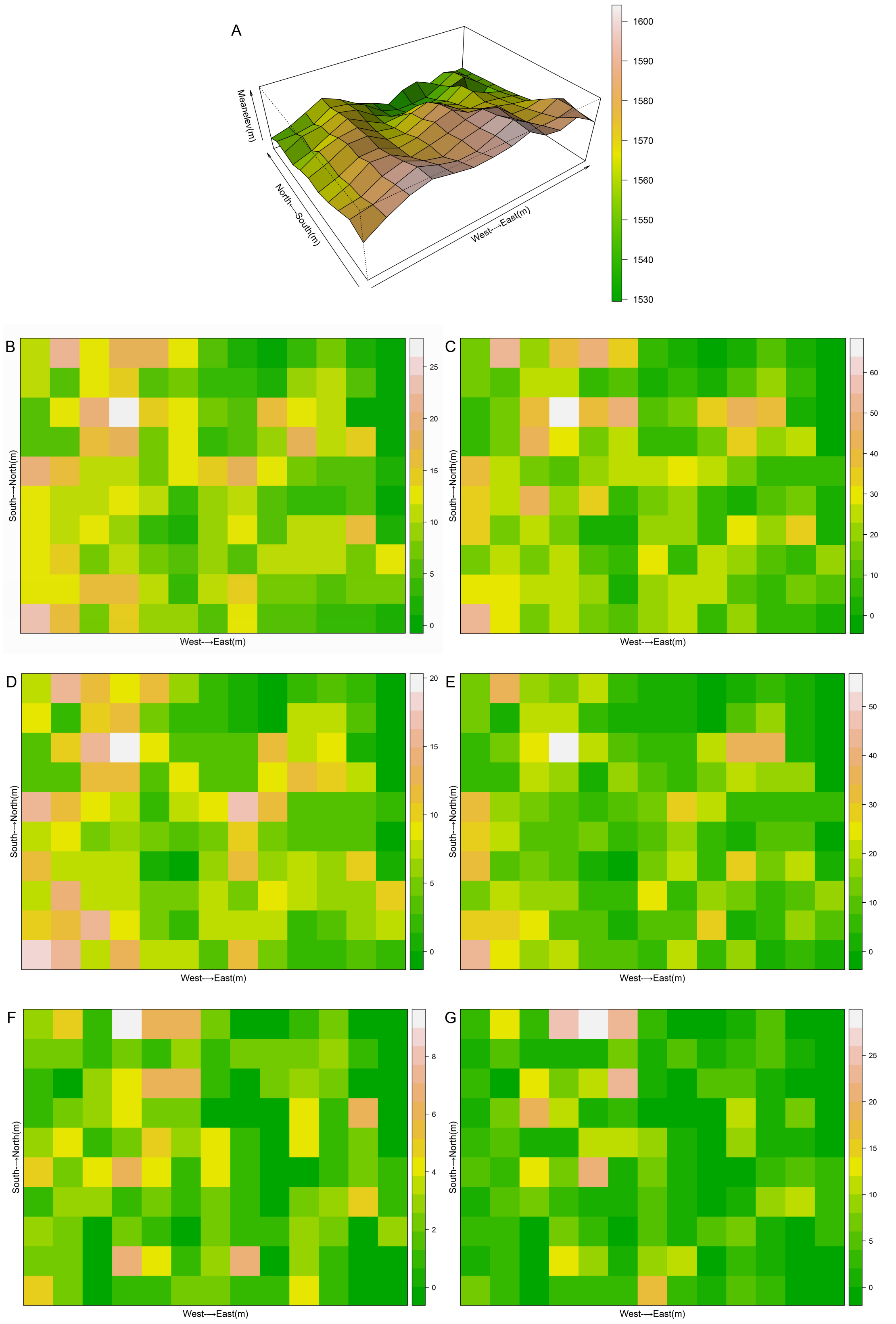

Fig.S2 Maps of the topography (A), overall fungi species richness (B), overall fungi species abundance (C), soil fungi species richness (D), soil fungi species abundance (E), rotten-wood fungi species richness (F), and rotten-wood fungi species abundance (G) at the scale of 20 m × 20 m in the 5 ha forest plot.

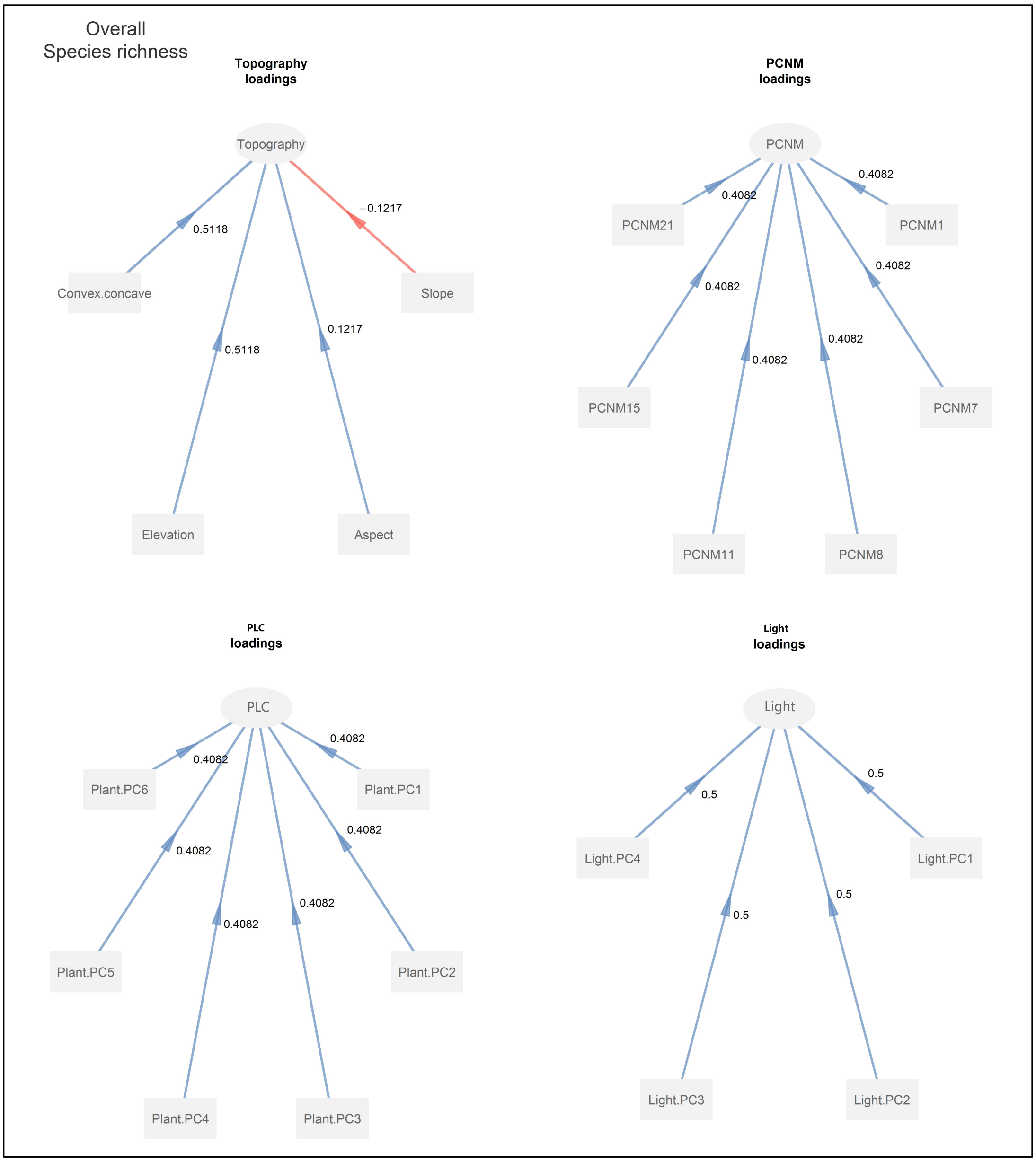
Overall
Species richness
Topography
loadings
Topography
0.5118
−0.1217
Convex.concave
Slope
0.5118
0.1217
Elevation
Aspect
PCNM
loadings
PCNM
0.4082
PCNM21
PCNM1
0.4082
0.4082
0.4082
PCNM15
PCNM7
0.4082
0.4082
PCNM11
PCNM8
PLC
loadings
PLC
0.4082
Plant.PC6
Plant.PC1
0.4082
0.4082
0.4082
Plant.PC5
Plant.PC2
0.4082
0.4082
Plant.PC4
Plant.PC3
Light
loadings
Light
0.5
Light.PC4
Light.PC1
0.5
0.5
0.5
Light.PC3
Light.PC2

38

39 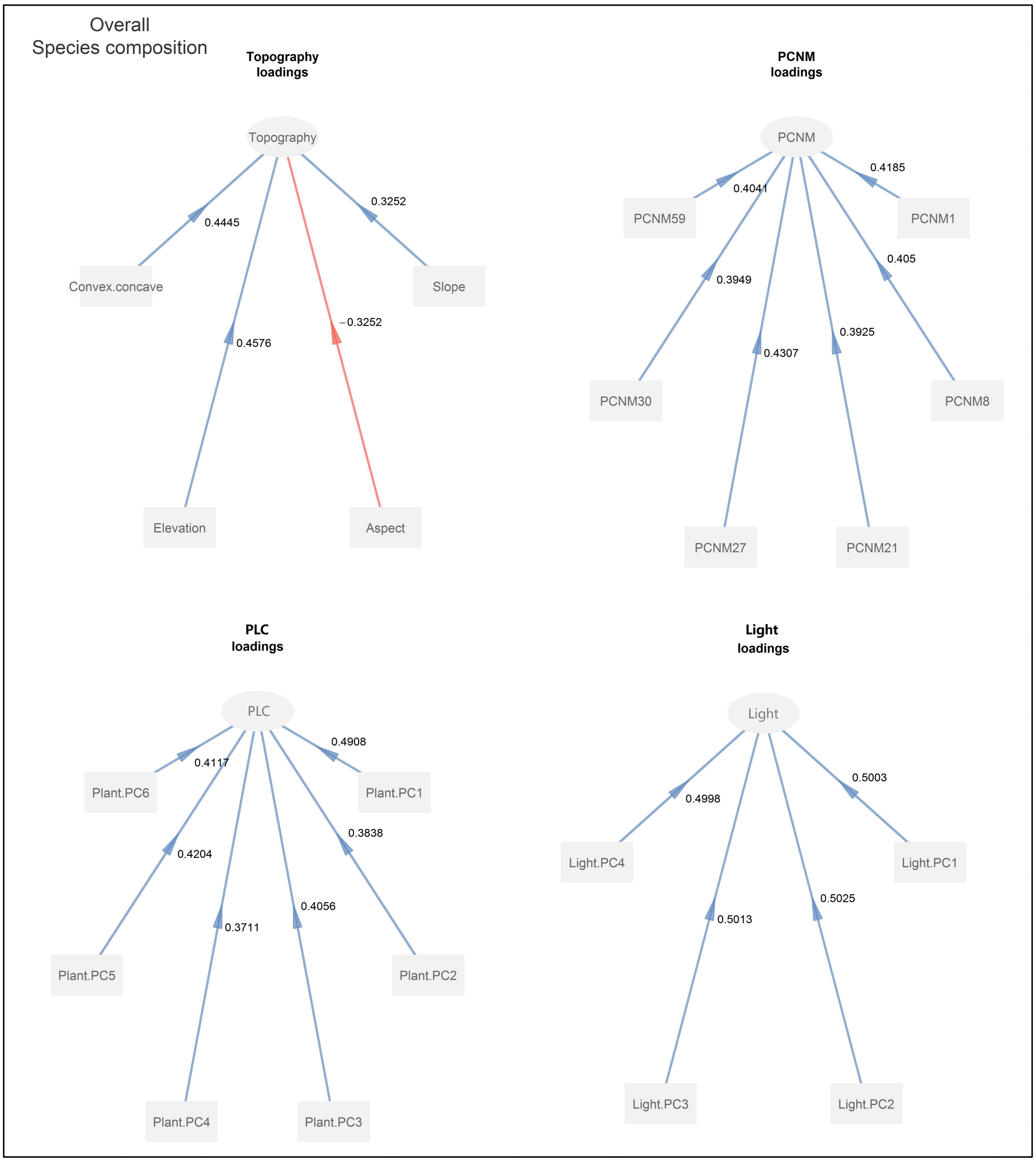
Overall
Species composition
Topography loadings
Topography
0.4445
0.3252
Convex.concave
Slope
0.4576
−0.3252
Elevation
Aspect
PCNM loadings
PCNM
0.4185
0.4041
PCNM59
PCNM1
0.405
0.3949
0.3925
0.4307
PCNM30
PCNM8
PCNM27
PCNM21
PLC loadings
PLC
0.4908
0.4117
Plant.PC6
Plant.PC1
0.3838
0.4204
0.4056
0.3711
Plant.PC5
Plant.PC2
Plant.PC4
Plant.PC3
Light loadings
Light
0.5003
0.4998
Light.PC4
Light.PC1
0.5025
0.5013
Light.PC3
Light.PC2

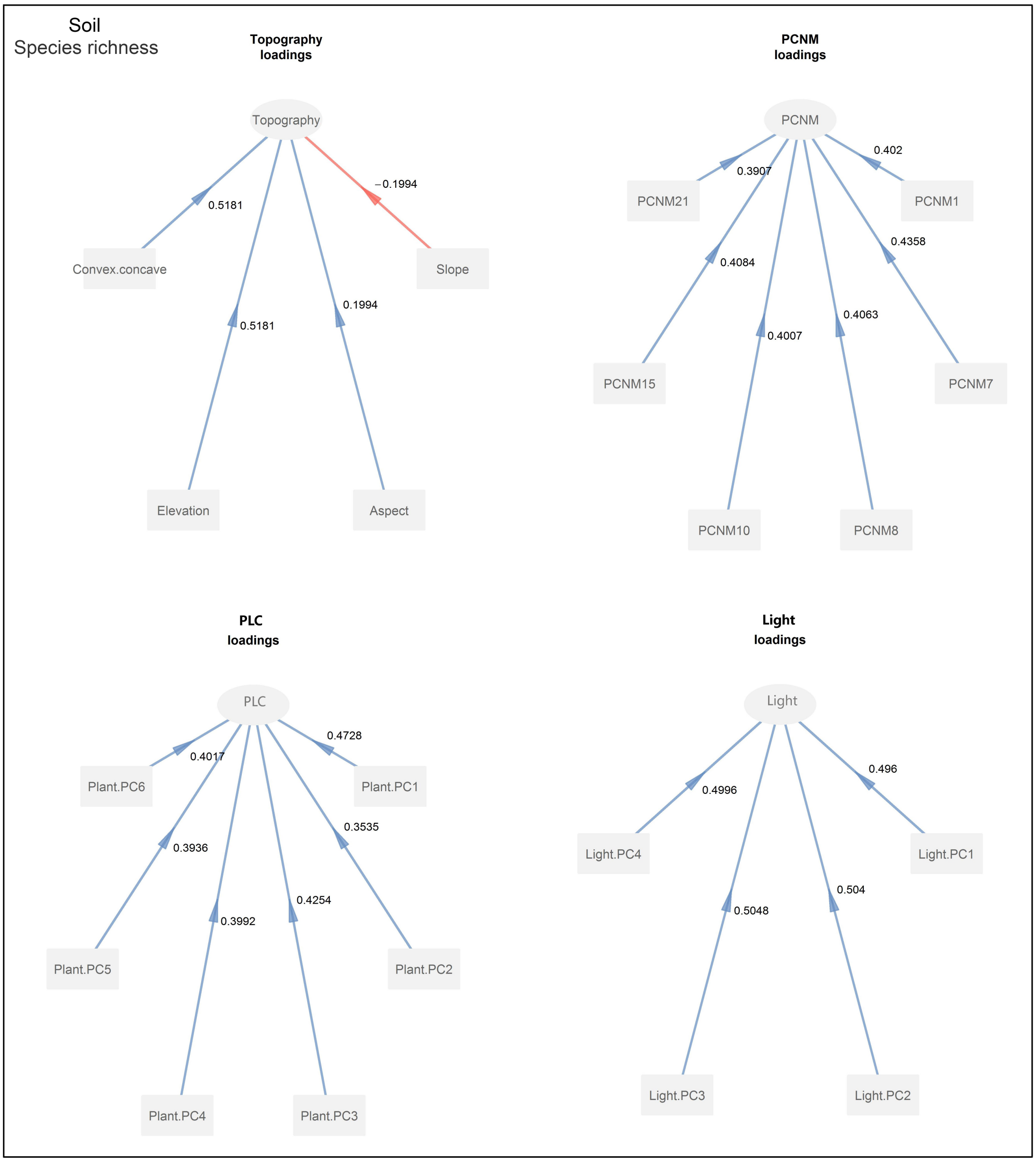
Soil
Species richness
Topography loadings
Topography
0.5181
−0.1994
Convex.concave
Slope
0.5181
0.1994
Elevation
Aspect
PCNM loadings
PCNM
0.402
0.3907
PCNM21
PCNM1
0.4358
0.4084
0.4063
0.4007
PCNM15
PCNM7
PCNM10
PCNM8
PLC loadings
PLC
0.4728
0.4017
Plant.PC6
Plant.PC1
0.3535
0.3936
0.4254
0.3992
Plant.PC5
Plant.PC2
Plant.PC4
Plant.PC3
Light loadings
Light
0.496
0.4996
Light.PC4
Light.PC1
0.504
0.5048
Light.PC3
Light.PC2

41

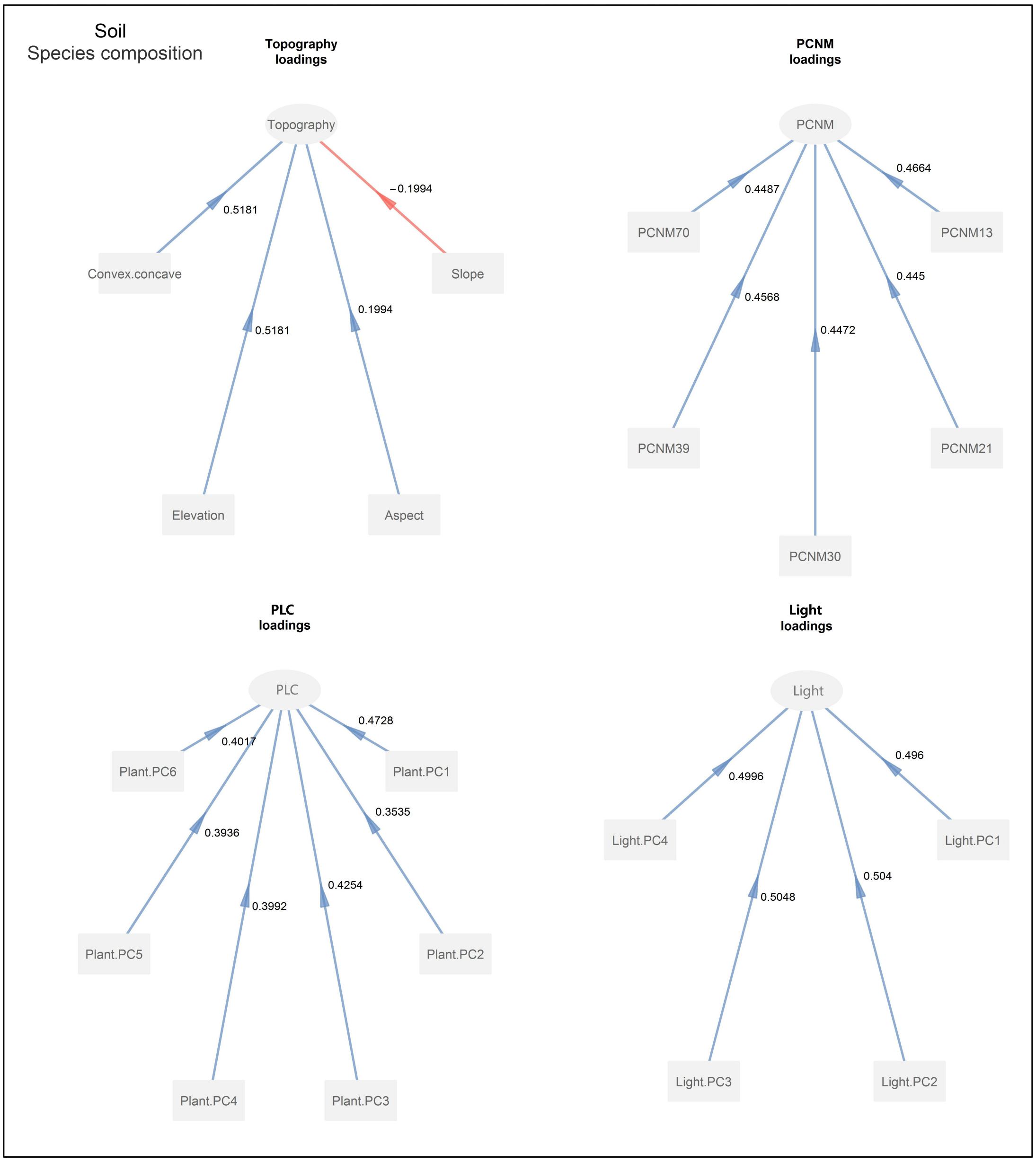
Soil
Species composition
Topography loadings
Topography
0.5181
−0.1994
Convex.concave
Slope
0.5181
0.1994
Elevation
Aspect
PCNM loadings
PCNM
0.4487
0.4664
PCNM70
PCNM13
0.4568
0.445
0.4472
PCNM39
PCNM21
PCNM30
PLC loadings
PLC
0.4017
0.4728
Plant.PC6
Plant.PC1
0.3936
0.3535
0.3992
0.4254
Plant.PC5
Plant.PC2
Plant.PC4
Plant.PC3
Light loadings
Light
0.4996
0.496
Light.PC4
Light.PC1
0.5048
0.504
Light.PC3
Light.PC2

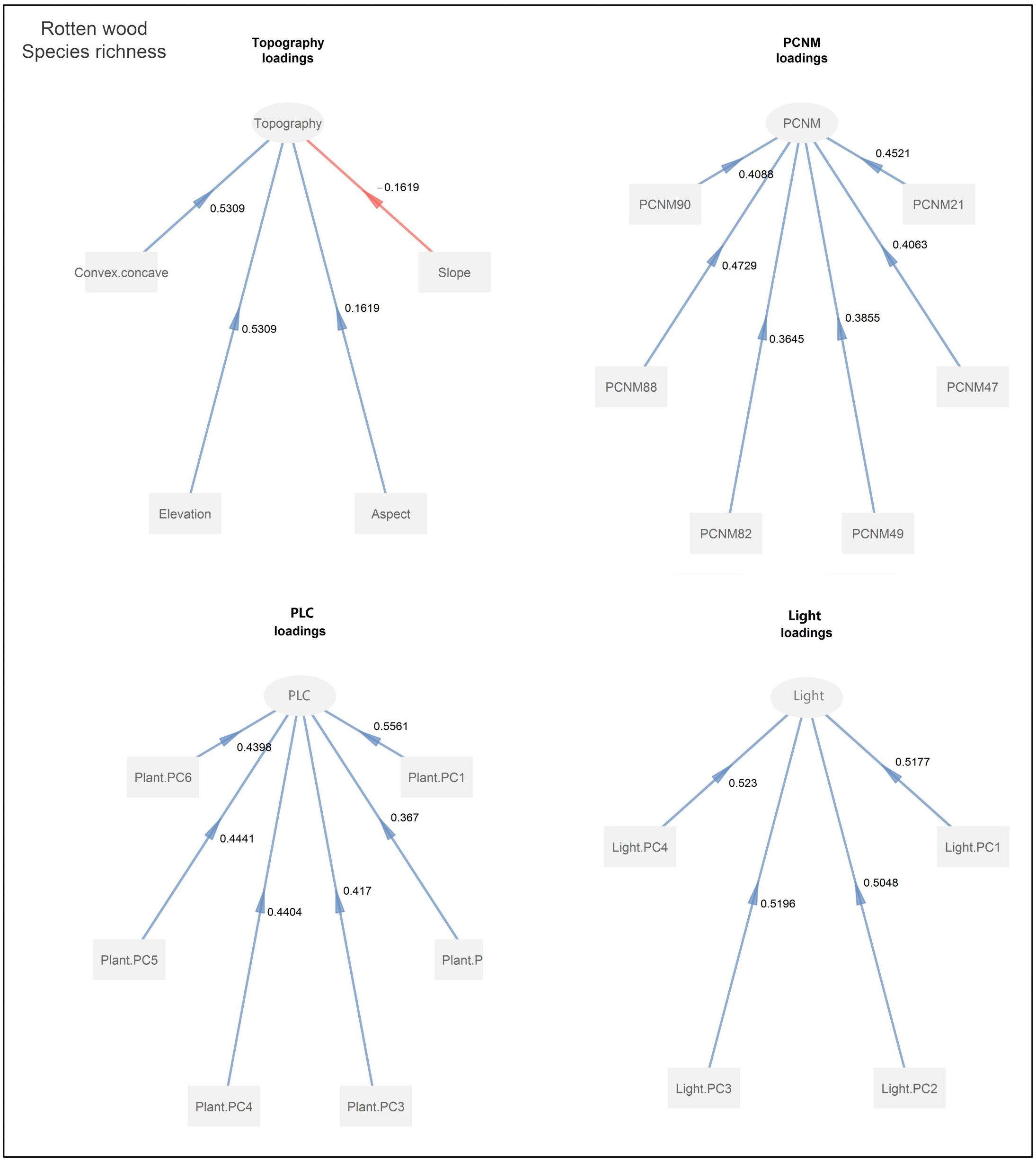
Rotten wood
Species richness
Topography
loadings
Topography
0.5309
Convex.concave
0.5309
Elevation
0.1619
Aspect
−0.1619
Slope
PCNM
loadings
PCNM
PCNM90
0.4088
0.4521
PCNM21
0.4729
0.4063
PCNM88
0.3645
0.3855
PCNM47
PCNM82
PCNM49
PLC
loadings
PLC
Plant.PC6
0.4398
0.5561
Plant.PC1
0.4441
0.367
Plant.PC5
0.4404
0.417
Plant.P
Plant.PC4
Plant.PC3
Light
loadings
Light
0.523
Light.PC4
0.5177
Light.PC1
0.5196
0.5048
Light.PC3
Light.PC2

42

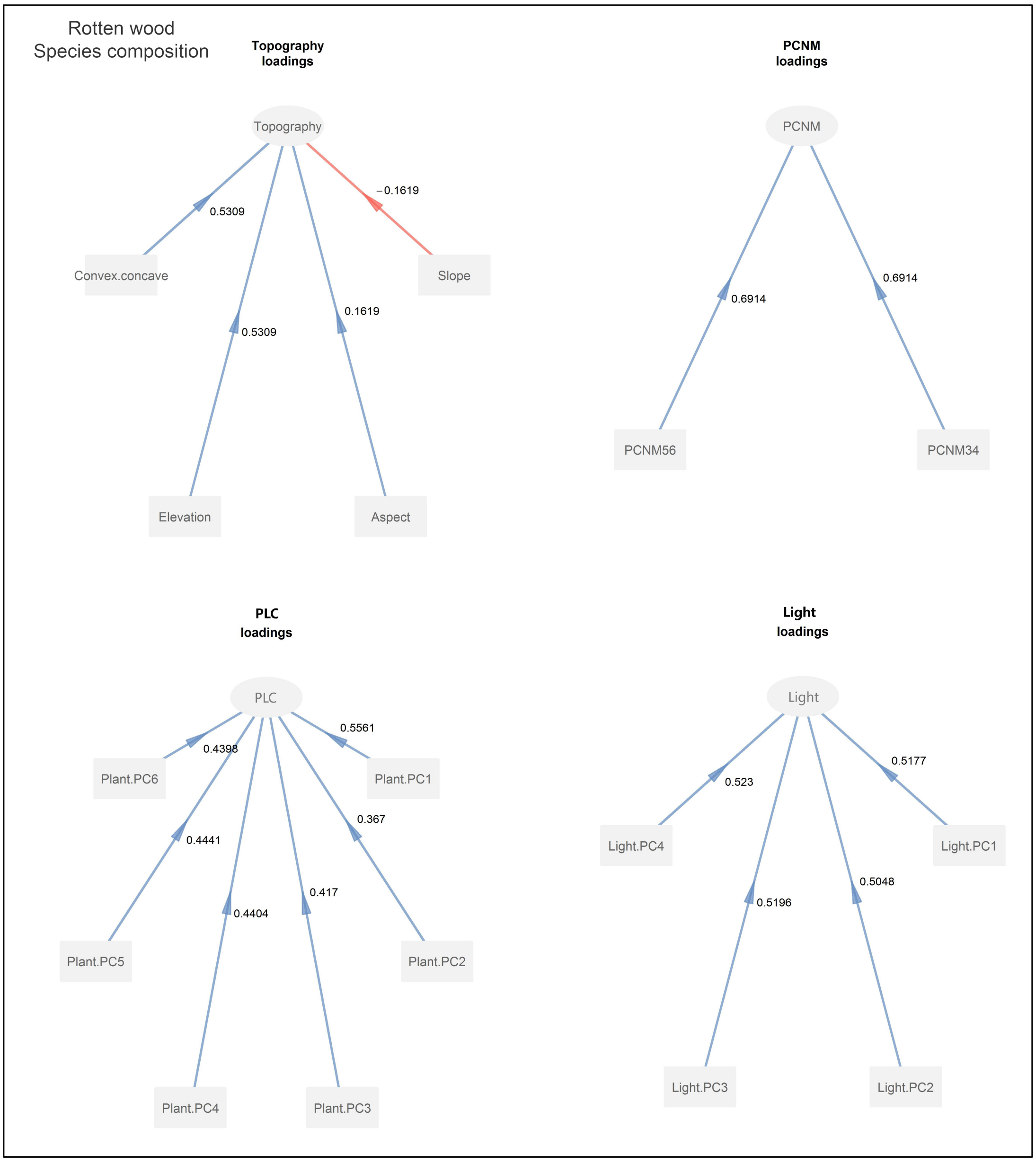

Fig. S3 Outer models (the relationship among indicators) of partial least square path modeling (PLS-PM). Spatial eigenvectors were obtained by principal coordinates of neighbor matrices (PCNM). Plant community: PLC; Plant PC1: basal area of *Quercus aliena* var. *acutiserrata*; Plant PC2: basal area of *Pinus armandii*; Plant PC3: basal area of *Pinus tabuliformis*; Plant PC4: basal area of *Toxicodendron vernicifluum* and *Euptelea pleiosperma*; Plant PC5: basal area of *Lindera obtusiloba* and *Sorbus alnifolia*; Plant PC6: basal area of *Corylus heterophylla*. Light PC1: canopy gap and radiation; Light PC2: canopy cover; Light PC3: light transmittance; Light PC4: scattered radiation. Blue and red lines indicate positive and negative pathways, respectively.
